# Supplementary material for: Design and Validation of Nanofibers Made of Self-Assembled Peptides to Become Multifunctional Stimuli-Sensitive Nanovectors of Anticancer Drug Doxorubicin
Source: Pharmaceutics. 2022 Jul 25;14(8):1544. doi: 10.3390/pharmaceutics14081544 (PMC9331957; doi:10.3390/pharmaceutics14081544)
Supplement: Supplementary file 1 [file pharmaceutics-14-01544-s001.zip › pharmaceutics-1815214-supplementary.pdf]

Article

Design and Validation of Nanofibers Made of Self-Assembled Peptides to Become Multifunctional Stimuli-Sensitive Nanovectors of Anticancer Drug Doxorubicin

Valentina Del Genio [1,2], Annarita Falanga [3], Emilie Allard-Vannier [2], Katel Hervé-Aubert [2], Marilisa Leone[4], Rosa Bellavita [1], Rustem Uzbekov [5], Igor Chourpa [2,*], Stefania Galdiero [1,*]

[1] Department of Pharmacy, School of Medicine, University of Naples 'Federico II', Via Domenico Montesano 49, 80131 Naples, Italy; valentina.delgenio@unina.it (V.D.G.); rosa.bellavita@unina.it (R.B.)
[2] EA 6295 Nanomédicaments et Nanosondes, Faculté de Pharmacie, Université de Tours, 31 Avenue Monge, 37200 Tours, France; emilie.allard@univ-tours.fr (E.A.-V.); katel.herve@univ-tours.fr (K.H.-A.)
[3] Department of Agricultural Science, University of Naples 'Federico II', Via Università 100, 80055 Portici, Italy; annarita.falanga@unina.it
[4] Institute of Biostructures and Bioimaging-CNR, 80145 Naples, Italy; marilisa.leone@cnr.it
[5] Plateforme Scientifique et Technique "Analyse des Systèmes Biologiques" (PST ASB), UFR de Médecine, 37032 Tours, France; rustem.uzbekov@univ-tours.fr
[6] Faculty of Bioengineering and Bioinformatics, Moscow State University, 119992, Moscow, Russia
* Correspondence: igor.chourpa@univ-tours.fr (I.C.); stefania.galdiero@unina.it (S.G.); Tel.: +39-081-2534503 (S.G.)

Table of contents

HPLC chromatograms and analytical data of peptides **P1, P2, P3** S2-S3

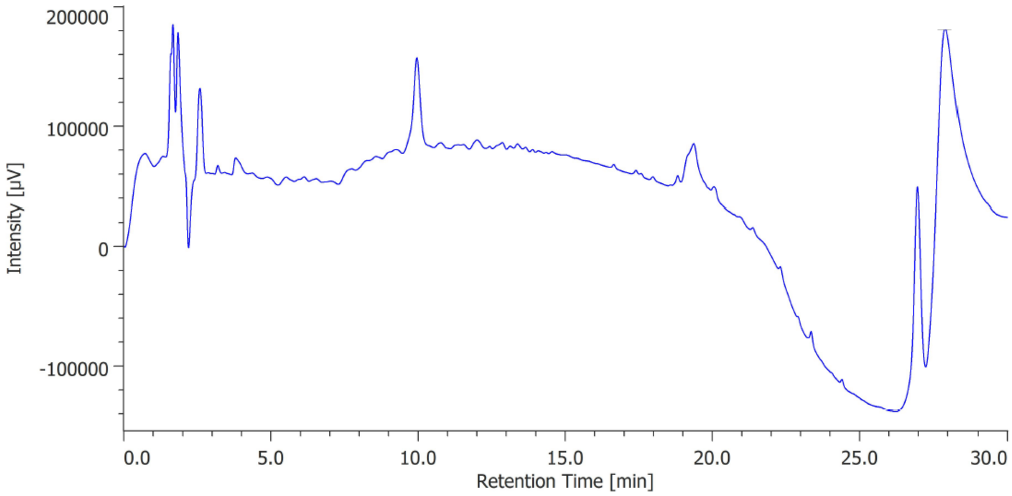

Figure S1. Chromatogram of peptide **P1** obtained by an analytical HPLC (Jasco LC-NetII/ADC) equipped with a Phenomenex Kinetex C18 column (150 mm × 4.6 mm, 5 µm, 100 Å), and monitored by UV detection at 220 nm. t_R: 9.955 min [linear gradient 20-80% MeCN (0.1% TFA) in H_2O (0.1% TFA) over 20 min, flow rate of 1 mL/min]. Calculated mass:1225.7, Found mass: $[M+H]^+$=1226.8

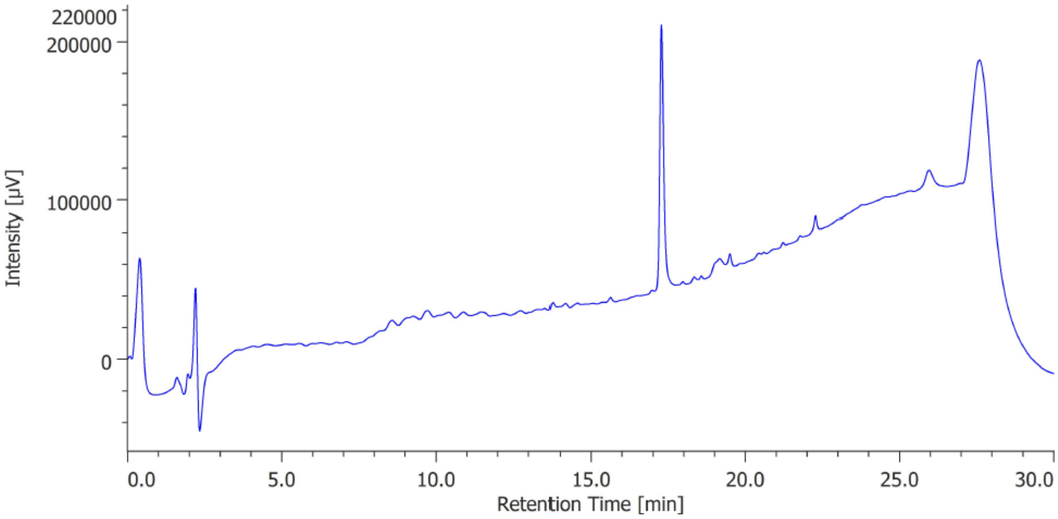

Figure S2. Chromatogram of peptide **P2** obtained by an analytical HPLC (Jasco LC-NetII/ADC) equipped with a Phenomenex Kinetex C18 column (150 mm × 4.6 mm, 5 µm, 100 Å), and monitored by UV detection at 220 nm. t_R: 17.272 min [linear gradient 10-90% MeCN (0.1% TFA) in H_2O (0.1% TFA) over 20 min, flow rate of 1 mL/min]. Calculated mass:1279.9, Found mass: $[M+2H]^+/2$=640.9.

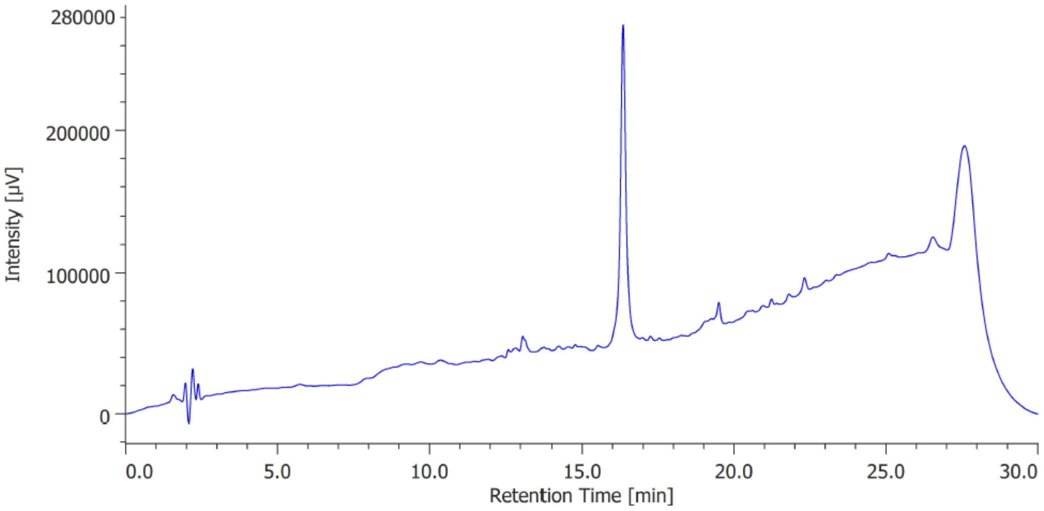

Figure S3. Chromatogram of peptide **P3** obtained by an analytical HPLC (Jasco LC-NetII/ADC) equipped with a Phenomenex Kinetex C18 column (150 mm × 4.6 mm, 5 µm, 100 Å), and monitored by UV detection at 220 nm. t_R: 16.338 min [linear gradient 10-90% MeCN (0.1% TFA) in H_2O (0.1% TFA) over 20 min, flow rate of 1 mL/min]. Calculated mass: 3559.1, Found mass: $[M+3H]^{3+}$ 1188.2, $[M+4H]^{4+}$ 891.5, $[M+5H]^{5+}$ 713.4.
